# Poorer mental health and sleep quality are associated with greater self-reported reward-related eating during pregnancy and postpartum: an observational cohort study

**DOI:** 10.1186/s12966-021-01124-9

**Published:** 2021-05-01

**Authors:** Grace M. Betts, Leah M. Lipsky, Chelsie D. Temmen, Anna Maria Siega-Riz, Myles S. Faith, Tonja R. Nansel

**Affiliations:** 1 Social and Behavioral Sciences Branch, Division of Intramural Population Health Research, Eunice Kennedy Shriver National Institute of Child Health and Human Development, 6710B Rockledge Dr., MSC 7004, Bethesda, MD 20892 USA; 2 Departments of Nutrition and Biostatistics and Epidemiology, School of Public Health and Health Sciences, University of Massachusetts, 109 Arnold House, 715 Pleasant St, Amherst, MA 01003-9303 USA; 3 Department of Counseling, School, and Educational Psychology, Graduate School of Education, 420 Baldy Hall, University at Buffalo – SUNY, Buffalo, NY 14250-1000 USA

**Keywords:** Depressive symptoms, Perceived stress, Mental health, Sleep quality, Reward-related eating, Pregnancy, Postpartum

## Abstract

**Background:**

Depression, stress, and poor-quality sleep are common during pregnancy and postpartum, but the relationship of these factors with reward-related eating is not well understood. This observational cohort study examines associations of depression, stress, and sleep quality with self-reported reward-related eating in pregnancy and postpartum**.**

**Methods:**

Participants were enrolled at < 12 weeks gestation and followed through 1 year postpartum. Self-reported measures obtained at baseline and 23–31 weeks postpartum included the Edinburgh Postnatal Depression Scale, Perceived Stress Scale, Pittsburgh Sleep Quality Index; reward-related eating measures included the Power of Food Scale (assessing hedonic hunger), modified Yale Food Addiction Scale (assessing addictive-like eating), and frequency and intensity of cravings. Linear and logistic regression models estimated associations of depressive symptoms, stress, and sleep quality with reward-related eating during pregnancy and postpartum, as well as change in each predictor with change in outcome.

**Results:**

During pregnancy, greater depressive symptoms (β ± SE = 0.03 ± 0.01, ***p*** < .01), higher stress (0.03 ± 0.01, ***p*** < .01), and worse sleep quality (0.03 ± 0.01, ***p*** = 0.03) were associated with greater hedonic hunger. Similarly, greater depressive symptoms (OR = 1.08, 95% CI: 1.02, 1.14, ***p*** = .01), higher stress (OR = 1.09, 95% CI: 1.04, 1.14, ***p*** = <.01), and worse sleep quality (OR = 1.09, 95% CI: 1.00, 1.18, ***p*** = .04) were associated with greater odds of addictive-like eating. These associations were also significant in postpartum except that sleep quality was not associated with hedonic hunger. Greater depressive symptoms (β ± SE = 0.06 ± 0.02, ***p*** < .01; 0.08 ± 0.02, ***p*** = <.01), higher stress (0.04 ± 0.01, ***p*** < .01; 0.06 ± 0.02, ***p*** < .01), and worse sleep quality (0.11 ± 0.03, ***p*** < .01; 0.13 ± 0.03, ***p*** < .01) during pregnancy were associated with stronger and more frequent cravings, respectively. Increased depressive symptoms from pregnancy to postpartum was associated with increased hedonic hunger (β ± SE = 1.17 ± 0.57, ***p*** = 0.01) and addictive-like eating (0.88 ± 0.33, ***p*** = 0.01), and increased stress was associated with increased hedonic hunger (1.71 ± 0.76, ***p*** = 0.02). Change in stress was not associated with change in addictive-like eating and change in sleep quality was not associated with change in either hedonic hunger or addictive-like eating.

**Conclusions:**

Greater depressive symptoms, perceived stress, and poorer sleep quality are associated with greater self-reported reward-related eating during pregnancy and postpartum, suggesting that efforts to improve diet during and after pregnancy may benefit from addressing mental health and sleep.

**Trial registration:**

Clinicaltrials.gov

Registration ID – NCT02217462.

Date of registration – August 13, 2014

**Supplementary Information:**

The online version contains supplementary material available at 10.1186/s12966-021-01124-9.

## Background

On average, pregnant women do not meet dietary recommendations [1]. Understanding neurobehavioral influences on maternal diet quality may have important implications for improving maternal [2, 3] and infant health outcomes [4–6]. In non-pregnant adults greater self-reported sensitivity to reward is associated with more intense and frequent food cravings [7], higher BMI [7, 8], and emotional overeating [8]. Additionally, greater self-reported reward sensitivity is associated with greater activation in reward-related brain areas in response to appetizing food images [9]. Population-based studies have shown positive associations of self-reported reward-related eating with intake of discretionary foods [10] and BMI [11]. Although food cravings are widely reported during pregnancy and are positively associated with discretionary food intake and excessive gestational weight gain [12], little is known about the correlates of reward-related eating in pregnant and postpartum women.

Depression [13, 14], stress [15–17], and insufficient sleep [18] are common during pregnancy and postpartum [13, 16, 18]. Furthermore, neuroimaging studies of nonpregnant adults indicate these factors can impact the functioning of neural circuits known to regulate reward-seeking behavior, motivation, and eating [19–21]. Depressive symptoms are associated with changes in blood flow in brain areas related to motivation, emotion, and appetite [20], structural and functional alterations within the brain’s reward circuitry [22], as well as blunted neural response to reward [23]. These alterations have been posited to explain associations of depression with poor food selection (e.g., lower intake of fruits and vegetables and higher intake of fast food and soda) [24, 25], undereating, and compensatory overeating of highly rewarding foods [26]. Depressive symptoms have also been associated with greater motivation to seek out food reward [27] and are significantly higher in individuals with addictive-like eating [28–30]. However, few prospective studies have examined these associations, and none have investigated these relations in pregnant or postpartum women.

Stress may also impact brain reward response and reward-related eating behaviors [21]. Acute stress has been associated with lower neural activity in response to monetary rewards [21] and food rewards [31]. Perceived stress has been associated with greater emotional eating and lower eating competence in overweight women [32], and greater consumption of sweets and salty snacks and lower consumption of fruits and vegetables in healthy adults [33, 34]. Additionally, findings from the Nurses’ Health study indicate that addictive-like eating is more prevalent in women with a history of Post-Traumatic Stress Disorder (PTSD) [35] or child abuse [36].

Consistent evidence from experimental neuroimaging studies of nonpregnant healthy adults indicates that sleep deprivation enhances the neural response to food reward [37–39] and non-food reward [19]. Additionally, observational studies show that insufficient sleep is also associated with increased energy intake in healthy men and women [40] and increased portion size selection in healthy women [41]. There are likely multiple pathways explaining this association, one hypothesis being that insufficient sleep is associated with greater sensitivity to food reward [42]. However, relationships of stress and sleep with self-reported reward-related eating have not been examined.

The purpose of this study was to address these knowledge gaps by investigating relationships of reward-related eating with depression, stress, and sleep quality in pregnant and postpartum women. We hypothesized that more depressive symptoms, higher perceived stress, and worse sleep quality are associated with higher self-reported reward-related eating during pregnancy and postpartum.

## Methods

### Participants and procedures

Data are from the Pregnancy Eating Attributes Study (PEAS), a longitudinal observational study of women from early pregnancy (< 12 weeks gestation) to 1 year postpartum [43]. Participants (*n* = 458) were recruited while obtaining prenatal care at University of North Carolina (UNC) at Chapel Hill Women’s Hospital. Written informed consent was obtained at recruitment. Enrollment began in October 2014 and continued through October 2016, with the following inclusion criteria: the mother (1) planned to deliver at UNC Women’s Hospital (singleton pregnancy expected) and remain in the area through 1 year postpartum, (2) had a BMI ≥18.5 kg/m2, (3) was between 18 and 45 years old, (4) had the ability to read and write in English, and (5) had access to internet and email. Women were excluded based on the following: multiple pregnancy, participant-reported psychiatric or eating disorder, or pre-existing diabetes or other medical condition contraindicating study participation. Self-report questionnaires were completed online within designated study windows at baseline (< 12 weeks gestation), 16–22 weeks, and 28–32 weeks gestation, and postpartum at 4–6 weeks, 6 months, and 12 months. Depressive symptoms, stress, sleep quality, and reward-related eating (addictive-like eating, hedonic hunger, and cravings) were assessed at baseline and 6 months postpartum; cravings and sociodemographic characteristics were assessed at baseline. In person study visits collected data on anthropometrics, and included the collection of biospecimens at various times. Study procedures were approved by the University of North Carolina Institutional Review Board.

### Measures

#### Depression

Depression was assessed using the 10-item Edinburgh Postnatal Depression Scale [44]. Example items include, “I have been able to laugh and see the funny side of things” with responses ranging from 0 (*as much as I always could*) to 3 (*not at all*). The scale demonstrated strong internal consistency during pregnancy (Cronbach’s alpha = 0.84) and postpartum (Cronbach’s alpha = .88). Total scores range from 0 to 30; higher scores indicate greater depressive symptoms.

#### Stress

Stress was assessed using the 10-item Perceived Stress Scale measuring perceived stress over the past month [45, 46]. Example items include, “In the last month, how often have you felt nervous and ‘stressed’?” with Likert-type responses ranging from 0 (*never*) to 4 (*very often*). The scale demonstrated strong internal consistency during pregnancy (Cronbach’s alpha = 0.89) and postpartum (Cronbach’s alpha = .91). Responses to each item are summed to produce a total score ranging from 0 to 40; higher scores indicate higher perceived stress.

#### Sleep quality

Sleep quality was assessed using the 9-item Pittsburgh Sleep Quality Index, which covers seven domains of sleep quality: subjective sleep quality, sleep latency, sleep duration, habitual sleep efficiency, sleep disturbances, use of sleep medication, and daytime dysfunction [47]. Items query sleep habits during the past month with most questions asking respondents to report how often they have had trouble sleeping for several reasons. Response options range from “not during the past month” to “three or more times a week.” The scale demonstrated strong internal consistency during pregnancy (Cronbach’s alpha = 0.74) and postpartum (Cronbach’s alpha = 75). Total scores range from 0 to 21; higher scores indicate poorer sleep quality.

#### Addictive-like eating

The 9-item modified Yale Food Addiction Scale measures symptoms of addictive-like eating [28]. Seven of the nine items ask participants to report frequency of addictive eating symptoms, such as “I feel sluggish or fatigued from overeating,” with responses ranging from 1 (*never*) to 5 (*≥ 4 times/week*). Based on the published threshold criterion responses for each symptom are dichotomized as below or meeting threshold. The remaining two items assess the presence of “clinically significant impairment,” e.g., “I kept consuming the same types or amounts of food despite significant emotional and/or physical problems related to my eating,” with response options *yes* or *no*. Responses to the 9 items were coded as 0 (not meeting threshold or negative response) or 1 (meeting threshold or positive response) and summed. Due to highly skewed distribution of responses, scores of ≥2 were collapsed into one group for cross-sectional pregnancy and postpartum analyses. This group accounted for 12% of participants, with 70% having a score of 0 and 18% a score of 1. For calculation of change scores, the full continuous range of scores was used.

#### Hedonic hunger

The 15-item Power of Food Scale [48] measures hedonic hunger, conceptualized as the psychological appetitive response to environmental food cues. Questions include, “I find myself thinking of food even when I’m not physically hungry,” and “If I see or smell a food I like, I get a powerful urge to have some,” with responses rated on a 5-point Likert scale from 1 (*don’t agree at all*) to 5 (*strongly agree*). The scale demonstrated strong internal consistency during pregnancy (Cronbach’s alpha = 0.88) and postpartum (Cronbach’s alpha = .92). Total scores range from 1 to 5 and are calculated as the mean of three component scores, which are calculated from the mean of the corresponding items. Higher scores indicate greater hedonic hunger.

#### Cravings

Food cravings were assessed using items developed by the study investigators, given the lack of an existing validated measure in pregnant women. The measure allowed participants to list their most craved foods, rather than responding to a predefined list, and enabled the assessment of both the frequency and intensity of overall and specific food cravings. One item asked, “In the past month, have you ever had a craving or strong desire to eat or drink a food or beverage such that you simply could not resist consuming the item?” If the answer was yes, participants listed up to three foods or beverages craved, and then rated the craving frequency, ranging from 1 *(never)* to 5 *(very frequently),* and strength, ranging from 1 *(mild)* to 5 *(strongest imaginable)*. Craving frequency and intensity were calculated as the highest response across foods.

#### Covariates

Variables hypothesized to be causally related to the predictors and outcomes of interest, based on a review of literature, were considered as confounders. Participants self-reported demographic information including age, marital status, education, household income, and household size at baseline. Marital status was categorized as either married or not married. Education was categorized as less than a bachelor’s degree, bachelor’s degree, or graduate degree. Income-poverty ratio was calculated as the ratio of household income relative to the poverty threshold according to household size [49] with higher values indicating greater income relative to the poverty threshold.

### Analysis

Data were analyzed using SAS software Version 9.4 [50]. Pearson correlations estimated bivariate associations among the main exposures and outcomes in pregnancy and postpartum, with the Sidak method used to adjust for multiple comparisons. General linear regression models were used to analyze continuous outcomes (hedonic hunger and cravings); ordered logistic regression models were used to analyze ordinal outcomes (addictive-like eating). Separate models were used to investigate relationships with each of the independent variables of interest (depressive symptoms, stress, and sleep quality) during pregnancy and during postpartum. Age, marital status, education, and income-to poverty ratio were included as covariates for all models. Paired t-tests examined changes in the main exposures and outcomes from early pregnancy to 6 months postpartum. General linear regression models examined whether change in each predictor (calculated by subtracting the pregnancy from the postpartum score) was associated with change in reward-related eating. Baseline scores for each variable were included in the models along with the covariates indicated above.

## Results

Of the 458 women enrolled, 91 (20%) withdrew prior to delivery and 46 (10%) withdrew or were lost to follow-up during postpartum. Women with available data on at least one predictor or outcome were included in the analysis (*N* = 373). Those included were on average 1.8 years older (1.78 ± 0.56, p = <.01) with a baseline BMI 2.9 points lower (− 2.93 ± 0.94, p = <.01) than those not included. The majority of the participants were over 30, non-Hispanic white, married, and had at least a bachelor’s degree (Table 1).

**Table 1:** Sample Demographic Characteristics

Variable	N	Mean ± SD or N (%)
Age	373	30.79 ± 4.58
Marital status	373	
Married		324 (91.8%)
Not married		29 (8.2%)
Education	353	
Less than a bachelor’s degree		93 (26.4%)
Bachelor’s degree		107 (30.3%)
Graduate degree		153 (43.3%)
Income-poverty ratio	350	3.92 ± 1.94
BMI	353	26.64 ± 6.55

As shown in Table 2, during pregnancy, depressive symptoms, stress, and sleep quality were each positively correlated with one or more measures of reward-related eating. Similar correlations were observed during postpartum, except that sleep quality was not correlated with either measure of reward-related eating. Depression (M ± SD of change = 0.52 ± 4.19), stress (0.80 ± 3.49), sleep quality (0.93 ± 3.49), and hedonic hunger (0.07 ± 0.52) increased from pregnancy to postpartum, but only the change in sleep quality (*p* < .001) was statistically significant. Addictive-like eating did not change from pregnancy to postpartum (− 0.02 ± 0.94, *p* = 0.71).

**Table 2:** Bivariate^a^ and descriptive statistics for pregnancy and postpartum

	1	2	3	4	5	6	7	*Pregnancy*		Postpartum		p^b^
								Mean (SD)	N	Mean (SD)	N	
1. Depressive Symptoms	**–**	.81**	.48**	**.22**	.24*	**–**	**–**	5.05 (4.11)	328	5.09 (4.44)	205	0.09
2. Stress	.62**	***–***	.44**	.29**	.27**	**–**	**–**	13.85 (6.25)	310	14.46 (6.67)	207	0.053
3. Sleep Quality	.50**	.44**	**–**	**.08**	**.22**	**–**	**–**	5.67 (3.22)	294	6.17 (3.41)	207	<.001
4. Hedonic Hunger	*.18*	.25**	.14*	**–**	.38**	**–**	**–**	2.19 (0.69)	353	2.25 (0.76)	227	0.06
5. Addictive-like Eating^c^	*.18*	*.18*	.20*	.31**	**–**	**–**	**–**	0.43 (0.70)	344	0.41 (0.70)	217	0.71
6. Cravings (Frequency)	*.18*	*.19*	.23**	.20*	*.07*	–	**–**	2.68 (1.51)	334	–	–	–
7. Cravings (Strength)	.21*	.23**	.24**	.22**	*.08*	.93**	–	1.95 (1.77)	334	–	–	–

*Note:* Italicized values are for pregnancy; bolded values are for postpartum. *p < .05; **p < .01

^a^Sidak method was used to adjust for multiple comparisons

^b^Paired t-test from pregnancy to postpartum

^c^Modified Yale Food Addiction Scale is used continuously here to reflect changes occurring above a score of 2

In regression analyses controlling for covariates, greater depressive symptoms, perceived stress, and worse sleep quality were associated with greater endorsement of hedonic hunger and addictive-like eating, and more frequent and intense cravings during pregnancy (Table 3). During postpartum, greater depressive symptoms and perceived stress were associated with greater endorsement of hedonic hunger and addictive-like eating. Worse sleep quality was associated with greater endorsement of addictive-like eating, but not with hedonic hunger.

**Table 3:** Associations of depression, stress, and sleep quality with reward-related eating and cravings

		Hedonic Hunger			Addictive-Like Eating			Cravings (Frequency)		Cravings (Strength)		
		*β (SE)*	*p*	*N*	*OR (95% CI)*	*p*	*N*	*β (SE)*	*p*	*β (SE)*	*p*	*N*
Pregnancy	Depressive Symptoms	0.03 (.01)	<.01	313	1.08 (1.02, 1.14)	.01	313	0.06 (0.02)	<.01	0.08 (0.02)	<.01	309
	Stress	0.03 (.01)	<.01	285	1.09 (1.04, 1.14)	<.01	298	0.04 (0.01)	<.01	0.06 (0.02)	<.01	274
	Sleep Quality	0.03 (.01)	.03	280	1.09 (1.00, 1.18)	.04	278	0.11 (0.03)	<.01	0.13 (0.03)	<.01	277
Postpartum	Depressive Symptoms	0.03 (.01)	.01	195	1.11 (1.03, 1.19)	<.01	193	–	–	–	–	
	Stress	0.03 (.01)	<.01	197	1.09 (1.04, 1.15)	<.01	195	–	–	–	–	
	Sleep Quality	0.02 (.02)	.35	199	1.13 (1.03, 1.23)	.01	196	–	–	–	–	

*Note:* Unstandardized estimates are reported. Linear and ordered logistic regression models adjusted for age, marital status, education, and income-to poverty ratio

Depressive symptoms, perceived stress and hedonic hunger were modestly higher in postpartum than pregnancy, although differences were not statistically significant (Table 2). Change in depressive symptoms from pregnancy to postpartum was positively associated with change in hedonic hunger and addictive-like eating (Table 4). Change in perceived stress was positively associated with change in hedonic hunger, but not addictive-like eating, and change in sleep quality was not associated with change in hedonic hunger or addictive-like eating.

**Table 4:** Associations of change in depression, stress, and sleep quality with change in reward-related eating

	∆ Hedonic Hunger			∆ Addictive-Like Eating		
	β (SE)	p	N	β (SE)	p	N
∆ Depressive Symptoms	0.02 (0.01)	.04	182	0.04 (0.02)	.01	180
∆ Stress	0.02 (0.01)	.02	183	0.02 (0.01)	.07	179
∆ Sleep Quality	−0.002 (0.01)	.86	168	0.009 (0.02)	.68	167

*Note:* Unstandardized estimates are reported. Linear regression models adjusted for baseline predictor score, age, marital status, education, and income-to poverty ratio

## Discussion

In this cohort of women followed from early pregnancy through postpartum, greater depressive symptoms, higher stress, and worse sleep quality were cross-sectionally associated with greater reward-related eating during pregnancy, as indicated by addictive-like eating, hedonic hunger, and the strength and frequency of cravings. Findings were similar during postpartum, except that sleep quality was not associated with hedonic hunger. In prospective analyses, changes in depressive symptoms and stress from pregnancy to postpartum were associated with changes in some indicators of reward-related eating. In contrast, sleep quality decreased from pregnancy to postpartum, but this change was not associated with change in reward-related eating. Overall, depressive symptoms and stress were cross-sectionally and prospectively associated with reward-related eating, while sleep quality was associated with reward-related eating only in cross-sectional analyses. Effect sizes were small but consistent across models, suggesting that these constructs are among multiple drivers of reward-related eating.

The positive associations of depressive symptoms and stress with reward-related eating and cravings observed in this study are consistent with previous studies in nonpregnant samples. Greater depressive symptoms [28–30] and trauma-related stress [35, 36] were associated with greater addictive-like eating as measured by the Yale Food Addiction Scale. Stress and psychological distress have also been associated with lower intake of fruits and vegetables and higher intake of high-fat and high-sugar foods in experimental and observational studies [24, 25]. Further, cravings were positively associated with anxiety and dysphoric mood [51] and the severity of depression [52]. Neuroimaging research shows that depressive symptoms are associated with blunted neural reward response [23], alterations within the brain’s reward circuitry [22], and changes in blood flow to areas of the brain related to motivation and appetite [20], providing a plausible physiological mechanism for associations of depressive symptoms with reward-related eating. A subset of women in the PEAS cohort participating in a focus group sub-study during their second trimester indicated that positive and negative moods often preceded food cravings [53], consistent with the current quantitative findings. The positive associations of depressive symptoms and stress with reward-related eating found in the present study are consistent with the hypothesis that reward-seeking is a compensatory behavior for the blunted neural response to reward that occurs in depression [26] and under stressed conditions [21, 31].

As reported herein, in prospective analyses, increased depressive symptoms from pregnancy to postpartum was associated with increased hedonic hunger and addictive-like eating. In contrast, while stress was positively associated with addictive-like eating and hedonic hunger in cross-sectional analyses, increased stress was prospectively associated only with increased addictive-like eating. The longitudinal association of stress with addictive-like eating is consistent with those from a retrospective study of women finding associations of trauma-related stress in childhood with addictive-like eating in adulthood [35, 36]. The null prospective association of stress with hedonic hunger suggests that the positive cross-sectional association could be due to confounding. Intervention studies are needed to clarify whether reducing depressive symptoms or stress impacts reward-based eating behaviors.

In this study, worse sleep quality was cross-sectionally associated with greater hedonic hunger, addictive-like eating, and food cravings during pregnancy, and greater addictive-like eating, but not hedonic hunger, during postpartum. These findings are largely consistent with previous research in nonpregnant samples finding positive associations of shorter sleep duration with food cravings [41, 54], appetite or hunger for energy-dense foods [41, 55], and neural activation in response to food stimuli, especially high-energy foods [37–39]. They are also consistent with research demonstrating that sleep restriction increases circulating concentrations of endocannabinoids (which are involved in the control of appetite and energy homeostasis) and concomitant reports of hunger and appetite [56]. The lack of association between sleep quality and hedonic hunger during postpartum suggests the relationship may be impacted by pregnancy status, and merits further investigation. It may also reflect the differences in the severity of reward-related eating represented by measures of addictive-like eating and hedonic hunger. Despite significant cross-sectional associations, and declines in sleep quality from pregnancy to postpartum, change in sleep quality was not prospectively associated with change in hedonic hunger or addictive-like eating. The lack of a prospective association could be due to the large proportion (40%) of women in this study whose sleep quality either did not change or changed by only 1 point from pregnancy to postpartum, which limits the power to detect associations between changes in sleep quality and reward-related eating.

Interpretation of these findings should consider the strengths and limitations of this study. Participants were recruited from a single location in the Southeastern United States with limited racial/ethnic and socioeconomic diversity, potentially limiting generalizability. However, the sample reflects the geographical area that is majority white with a bachelor’s degree or higher and with a median household income of $68,640 [57]. While sleep was assessed using self-report measures that are more susceptible to response bias than objective measures, the large sample size, measurement of and adjustment for covariates, as well as the use of validated measures of sleep quality, perceived stress, and depressive symptoms, and multiple self-report measures of reward-related eating strengthen the internal validity of the findings. Additionally, the prospective study design precludes recall bias and provides evidence for a temporal relationship between the variables of interest.

## Conclusions

In conclusion, depressive symptoms, perceived stress, and sleep quality were associated with multiple measures of reward-related eating during pregnancy and postpartum. Increased depressive symptoms and stress were associated with increased reward-related eating from pregnancy to postpartum, but no association was observed with change in sleep quality. Given the associations of reward-related eating with unhealthy food choices [58, 59] and increased intake of discretionary foods [10, 60, 61], these findings suggest the need to examine whether targeting mental health and sleep quality improves eating behaviors in women during pregnancy and postpartum. Assessing sleep quality and mental health may assist healthcare professionals counseling women on healthful eating.

## Supplementary Information

**Additional file 1.**

**Additional file 2.**

## Data Availability

The datasets used and/or analyzed during the current study are available from the corresponding author on reasonable request. Following publication of study objectives, de-identified data will be shared in the NICHD Data and Specimen Hub.

## Abbreviations

PEAS: Pregnancy Eating Attributes Study
BMI: Body mass index
