# Supplementary material for: Poorer mental health and sleep quality are associated with greater self-reported reward-related eating during pregnancy and postpartum: an observational cohort study
Source: Int J Behav Nutr Phys Act. 2021 May 1;18:58. doi: 10.1186/s12966-021-01124-9 (PMC8088672; doi:10.1186/s12966-021-01124-9)
Supplement: Supplementary file 2 — Additional file 2. [file 12966_2021_1124_MOESM2_ESM.docx]

**How sample was recruited:**

Participants (n=458) were recruited while obtaining prenatal care at University of North Carolina (UNC) at Chapel Hill Women’s Hospital from November 2014 through October 2016.

**How representative sample was of target group:**

The sample reflects the geographical area that is majority white with a bachelor’s degree or higher and with a median household income of $68,640.

**How analysed sample differed from the recruited sample:**

The analyzed sample did not significantly differ from the recruited sample on any of the included variables (depressive symptoms, perceived stress, sleep quality, addictive-like eating, hedonic hunger, craving strength and frequency, income-poverty ratio, age, marital status, and education) during pregnancy or postpartum.

**How any missing data were handled:**

The authors used a complete case analysis.
